# A framework for a consistent and reproducible evaluation of manual review for patient matching algorithms

**DOI:** 10.1093/jamia/ocac175

**Published:** 2022-10-28

**Authors:** Agrayan K Gupta, Suranga N Kasthurirathne, Huiping Xu, Xiaochun Li, Matthew M Ruppert, Christopher A Harle, Shaun J Grannis

**Affiliations:** Indiana University, Indianapolis, Indiana, USA; Center for Biomedical Informatics, Regenstrief Institute, Indianapolis, Indiana, USA; Department of Family Medicine, Indiana University School of Medicine, Indianapolis, Indiana, USA; Black Dog Institute, University of New South Wales, Sydney, New South Wales, Australia; Department of Biostatistics, Indiana University School of Medicine, Indianapolis, Indiana, USA; Department of Biostatistics, Indiana University School of Medicine, Indianapolis, Indiana, USA; Department of Medicine, University of Florida Health, Gainesville, Florida, USA; Precision and Intelligent Systems in Medicine (PrismaP), University of Florida, Gainesville, Florida, USA; Department of Health Outcomes and Biomedical Informatics, College of Medicine, University of Florida, Gainesville, Florida, USA; Center for Biomedical Informatics, Regenstrief Institute, Indianapolis, Indiana, USA; Department of Family Medicine, Indiana University School of Medicine, Indianapolis, Indiana, USA

**Keywords:** *JAMIA*, manual review, gold standard, patient matching, matching algorithms

## Abstract

Healthcare systems are hampered by incomplete and fragmented patient health records. Record linkage is widely accepted as a solution to improve the quality and completeness of patient records. However, there does not exist a systematic approach for manually reviewing patient records to create gold standard record linkage data sets. We propose a robust framework for creating and evaluating manually reviewed gold standard data sets for measuring the performance of patient matching algorithms. Our 8-point approach covers data preprocessing, blocking, record adjudication, linkage evaluation, and reviewer characteristics. This framework can help record linkage method developers provide necessary transparency when creating and validating gold standard reference matching data sets. In turn, this transparency will support both the internal and external validity of recording linkage studies and improve the robustness of new record linkage strategies.

## INTRODUCTION

The specialization of healthcare services and the mobility of patient populations have led to fragmented and incomplete health data in the US Healthcare providers require comprehensive patient records to ensure the quality, accuracy, safety, and cost-effectiveness of care. Despite widespread adoption of health information systems, such as electronic health records, patient information is typically not compiled into a single longitudinal record. Instead, many patients’ complete healthcare record is held across multiple siloed clinical repositories.^1^ This fragmentation can compromise patient safety, lead to duplicated testing, and impact physician clinical decisions.^2^ For example, incomplete patient and medication data account for nearly half of all medication errors.^3^ Inconsistent data also drive hospital costs through inefficient care. Duplicate records cost an average of $1100 for repeated tests and delays in care, and over $800 per emergency department visit.^4^^,^^5^ One-third of rejected insurance claims are attributed to inaccurate patient identification, which costs the average hospital $1.5 million and the US healthcare system $6 billion annually.^5^ These limitations have led healthcare systems to invest millions in record linkage and data management.^6^^,^^7^

To create complete longitudinal patient records, there must be approaches to effectively integrating data for the same patient across information systems and organizations. A logical solution to efficiently match patient information would be a unique patient identifier (UPI). However, the United States is the last industrialized nation without such a system.^8^ Congress has barred funding for developing a UPI for over 2 decades, primarily citing privacy concerns.^9^ To fully remove the prohibition, Congress must vote to remove the ban, which may take several years along with time to research and implement an identifier system.

Thus, without a UPI, US hospitals rely on probabilistic and heuristic patient matching algorithms driven by patient demographic information, social security numbers, and other identifiers extracted from existing medical records. Despite advanced algorithms with strong predictive power, lack of data standardization, and incomplete patient information, hinder matching accuracy. Further, some studies have found 15%–37% of linkage algorithm links rejected by human review.^10^ To overcome these barriers, there must be high-quality and standardized reporting of data elements, and reproducible methods to evaluate algorithmic quality, including accuracy and potential for bias.

Currently accepted and peer-reviewed methodologies for record linkage, such as GUILD, describe each step of the linkage pathway and recommends methods to assess or account for linkage error; however, they fail to describe the manual review processes that provides the basis for adjudicating performance.^11–14^ Prior record linkage studies that included a manual review process failed to adequately describe each step.^15–17^ Therefore, creating more transparent and detailed approaches to conducting manual review in record linkage studies fills a key literature gap, and may improve data integration efforts for reducing healthcare costs, and improving quality and patient safety.

### Objective

We propose a novel robust framework for a consistent and reproducible evaluation of manual review gold standard data sets for measuring patient matching algorithm performance.

## METHODS

We describe recommended manual review reporting elements for consistent gold standard data set creation and evaluation (Table 1). Because steps may differ in individual studies, we describe a general approach, and then variations for each element.

**Table 1.** Steps 1–4 describe the recommended manual review reporting elements for preparing a gold standard data set and record pairs through data description, preprocessing, blocking, and sampling; steps 5–8 describe human training, adjudication processes, result analysis, and a description of software and reviewers

Reporting element	Description
1. Data set description	How a complete data set is collected and from where is it sourced. This may include type of data source, provenance, and population coverage.
2. Preprocessing: Field Selection and Data Standardization	How data fields are selected and standardized prior to any record pair selection for the matching software. This includes removing all nonvalid values, using agreed-upon formats, and inputting values when needed.
3. Record pair grouping	Processes, such as blocking, to group record pairs based on schemes to reduce computational complexity
4. Procedure for sampling and matching pairs	Methods to sample record pairs based on blocking schemas, and match them prior to human review
5. Training and process for judging record matches and nonmatches	Training and instructions provided to and used by reviewers to judge a record pair as a “match” or “non-match”. This includes how record pairs are assigned to reviewers, and criteria that reviewers use to judge matches and nonmatches. This also includes any iterative review or other steps taken to adjudicate discordance across reviews judging the same record pairs, and to determine the final match status.
6. Inter-rater reliability measures	Any inter-rater reliability metrics, such as Cohen’s Kappa, record pair discordance and overall matches/nonmatches, their values, and a description of how they are used in the review process.
7. Review software or tools used	Software, forms, or other support tools used to present record pairs to reviewers and for reviewers to record their adjudications.
8. Reviewer characteristics	Total number of reviewers, and ideally each reviewer’s age, gender, race, cultural background, and prior experience with clinical or public health data and record linkage.

### Data set descriptions

Designing a reproducible process for record linkage requires well-described data sets. Furthermore, external validation of a linkage algorithm requires standardized metadata descriptors on the data set and its origin. Record linkage algorithms have been tested on various data sets, including cancer patients,^18^ newborns,^19^ and indigenous tribes,^20^ all with differing data quality. Thus, the data source should be clearly described, whether it is from electronic health records, public health records, social security master files, clinical registry, etc, as differences in data sources may impact algorithms’ generalizability to other data sources or types.^13^^,^^21^ The data set’s quality can be described through its provenance, collection techniques, and variables measured. Additionally, studies should describe the quality of variables in terms of completeness and accuracy. Poor data quality may lead to the clustering of identifier errors, resulting in linkage errors with unmatched or misclassified records and potential selection bias.^22^

### Preprocessing: field selection and data standardization

This component contains 2 intermediate steps: field selection and data standardization.

First, a set of fields are selected to describe the individual characteristics of each record. Some data sets may contain a patient’s social security number, which can function as a unique identifier. In the absence of such an identifier, fields such as date-of-birth and last name can combine as quasi-identifiers to uniquely identify patients.^23^ Additionally, chosen fields should contain accurate and complete data.^10^ For example, the first-name field may be empty for many records, which may necessitate additional selected fields to ensure record uniqueness.

After appropriate field selection, an agreed-upon preprocessing method is needed to standardize records for accurate review, as address standardization alone can decrease unlinked records by up to 20%.^24^ Also, DOB fields should follow a consistent format, that is “MM/DD/YYYY”.^25^ Accepted standards such as the US Postal Service address definitions and Uniform Hospital Discharge Dataset currently exist but need greater usage to nationalize a standard format for data element capture.^26^^,^^27^

### Record pair grouping (blocking)

When matching 2 data sets, each record between the data sets must be compared to each other. Researches utilize blocking to subset a large data set into smaller groups by common attributes.^28^ Specific blocking schemes efficiently reduce the computational complexity of record comparison by increasing the proportion of true matches by only comparing records within each controlled blocking scheme. Ideal blocking fields have a high variety of values and high rates of completeness.^24^ However, blocking may reduce review accuracy by removing true matches if performed without proper discretion.^29^

### Procedure for sampling and matching pairs

Records from each blocking group must be sampled to create a representative training set and final data set for manual review. Depending on the blocking scheme, and size of the data set, studies may use a proportional random or stratified sampling method. Studies should accurately report the sampling methodology for reproducibility and mitigating bias. Using a representative sample across multiple dimensions such as culture, location, and age, will reduce biases in the record linkage manual review process as reviewers can make clearer adjudications between records pairs.^30^^,^^31^

#### Process for judging record matches and nonmatches

Studies should include a comprehensive overview of reviewer training. The manual review process begins with reviewer instruction, and includes steps to assess records, evaluate biases and results, and resolve discordance between reviewers. Experts should design and curate a training record linkage data set, along with a gold standard, for the chosen reviewers to train with. Of note, to verify natural language processors for radiology reports, researchers have similarly formed expert review teams to review reports and create a reference validation data set for algorithms.^32^^,^^33^

Afterwards, researchers should review matches and mismatches between each reviewer and the gold standard to measure discordance. If significant disagreement is present, reviewers may receive additional training. After training, reviewers will use the same software for both training and final annotation. If reviewers have differing record judgment, another annotator may be used as a tiebreaker,^33^ and discrepancies may be resolved in group discussion.^34^

#### Inter-rater reliability measures

Researchers should measure inter-rater reliability to understand variation between reviewers who are adjudicating record pair matches and nonmatches.^35^ In manual review studies, such aspects are typically measured through Cohen’s Kappa (2 raters) or Fleiss Kappa (an adaptation of Cohen’s for 3 or more raters) statistics. Cohen’s Kappa calculates a score between 0 and 1, with 1 as perfect agreement between reviewers, by comparing the observed agreement versus the probability of chance agreement. Other reliability measures include percent agreement, and Pearson’s R correlation coefficient, though they may poorly reflect the true discordance.^36^ In addition, algorithm performance may be assessed through positive predictive value and F1 scores.^21^

#### Review software or tools used

A description of the software used should provide its features for future reproduction. A basic software at a minimum should: (1) import, query, and tabulate data from different data sets; (2) match records across distinct data sets; and (3) record and store discordance results from manual review per reviewer. Ideal review software streamlines the review process to reduce possible biases and focus reviewer attention solely on matching records with an accessible user interface. For example, Link Plus, a CDC probabilistic record linkage software with manual review capabilities, and Febrl, an open-source linkage package, automatically sort and group records, and color-code blocking variable match status for review ease.^37^^,^^38^ Additionally, they instantly treat null values as empty data, and have designated keyboard shortcuts. Software should also display previously matched records under reviewed records, so reviewers can analyze previous patient information, such as former addresses and phone numbers for accurate judgment.

#### Reviewer characteristics

Ideally, studies should describe reviewer characteristics, including the total number of reviewers, age, gender, race, cultural background, and prior experience with clinical or public health data and record linkage research. Since assessing matches among record pairs often includes comparing individual names from different backgrounds and as names vary across demographic, social, and cultural dimensions, a lack of diversity on a review team may introduce bias in matching records and curating a gold standard dataset. Diverse teams are more likely to remain objective and examine facts with greater scrutiny.^39^ Thus, by reporting reviewer characteristics, researchers may help readers and users of the data set understand potential biases in the data set and in record linkage algorithms validated using the data sets, critical to creating robust and trustworthy record linkage approaches.

## DISCUSSION

Automated methods for record linkage are becoming increasingly important as healthcare systems have widely adopted electronic health records and federal interests advocate for accurate patent matching.^7^^,^^9^ A critical step in creating and validating patient matching algorithms is establishing a gold standard to evaluate such algorithms against. To create high-quality gold standard data sets, there must be a framework that facilitates transparent reporting of manual review processes, thereby enabling critical evaluation and comparison of methodologies.

This framework builds upon prior work to provide detailed guidelines for the reproducible evaluation of the manual review process for patient matching algorithms. A crucial component that was outlined, but not described in the prior literature that established gold standards for record linkage. Such reporting enhances rigor and reproducibility, and allows end-users to better evaluate external validity and potential biases in the gold standard data sets as well as matching algorithms created using the data sets. More generally, this framework will provide critical support to technology developers and healthcare organizations in developing a nationwide strategy and approach to patient matching.

Unlike current patient matching methods, this framework supports evidence-based practices, and therefore health IT policymakers should explore strategies to expand the evidence base for real-world matching system performance, and encourage more consistent approaches to data collection and standardization.^24^^,^^40^^,^^41^ The Department of Health and Human Services and the ONC have made recent efforts in standardizing patient matching. These included: Project US@ for unified industry-wide specification for addresses, US Core Data for Interoperability to standardize health data classes for national information exchange, and the development of patient demographic matching specifications with the Interoperability Standards Advisory.^42–44^ Such efforts are crucial, as research has shown last name and address standardization can improve record linkage accuracy by up to 8%.^24^

### Limitations

This framework has 2 main limitations. First, curating a gold standard through human review requires significant personnel and technical costs.^17^^,^^45^ However, a number of institutions have formed manual review teams supported by federal funding, which have not only made record linkage manual review more feasible, but a growing area of study.^15^^,^^46^ Second, while this framework details several key elements in reproducible manual review, we do not intend for it to be a final framework, but to set the stage for future work by establishing general guidelines. It must be applied on real-world data to determine its robustness in application and based on results can be brought to public consensus for widescale acceptance and systematic usage.

## CONCLUSION

This 8-point framework provides consistent guidelines for record pair manual review when creating gold standard data sets for assessing patient matching algorithm performance. Such reporting provides critical transparency and rigor that is important to trustworthy and unbiased record linkage technology in the United States. Moreover, this framework provides new methods that will support healthcare organizations and policymakers in developing nationwide strategy for data integration efforts critical to reducing healthcare costs and improving quality.

## FUNDING

This work was supported by Agency for Healthcare Research and Quality grant number 5R01HS023808-04.

## AUTHOR CONTRIBUTIONS

SJG, CAH, and SNK contributed to the conception, design, acquisition, and analysis for the work. HX and XL performed analysis and contributed to design. MMR contributed to conception and design. AKG drafted the initial manuscript.

## CONFLICT OF INTEREST STATEMENT

None declared.

## Data Availability

No new data were generated or analyzed in support of this research.
